# Immune-Mediated Mechanisms of Action of Probiotics and Synbiotics in Treating Pediatric Intestinal Diseases

**DOI:** 10.3390/nu10010042

**Published:** 2018-01-05

**Authors:** Julio Plaza-Díaz, Francisco Javier Ruiz-Ojeda, Mercedes Gil-Campos, Angel Gil

**Affiliations:** 1 Department of Biochemistry and Molecular Biology II, School of Pharmacy, University of Granada, 18071 Granada, Spain; fruizojeda@ugr.es; 2 Institute of Nutrition and Food Technology “José Mataix”, Biomedical Research Center, University of Granada, Armilla, 18016 Granada, Spain; 3 Instituto de Investigación Biosanitaria ibs GRANADA, Complejo Hospitalario Universitario de Granada, 18014 Granada, Spain; 4 CIBEROBN (CIBER Physiopathology of Obesity and Nutrition CB12/03/30028), Instituto de Salud Carlos III, 28029 Madrid, Spain; mercedes_gil_campos@yahoo.es; 5 Pediatric Research and Metabolism Unit, Reina Sofia University Hospital, Maimonides Institute for Biomedical Research (IMIBIC), Av. Menendez Pidal s/n, 14010 Córdoba, Spain

**Keywords:** probiotics, pediatric gastrointestinal infection, mechanism of action, intestinal microbiota, immune system

## Abstract

The pediatric population is continually at risk of developing infectious and inflammatory diseases. The treatment for infections, particularly gastrointestinal conditions, focuses on oral or intravenous rehydration, nutritional support and, in certain case, antibiotics. Over the past decade, the probiotics and synbiotics administration for the prevention and treatment of different acute and chronic infectious diseases has dramatically increased. Probiotic microorganisms are primarily used as treatments because they can stimulate changes in the intestinal microbial ecosystem and improve the immunological status of the host. The beneficial impact of probiotics is mediated by different mechanisms. These mechanisms include the probiotics’ capacity to increase the intestinal barrier function, to prevent bacterial transferation and to modulate inflammation through immune receptor cascade signaling, as well as their ability to regulate the expression of selected host intestinal genes. Nevertheless, with respect to pediatric intestinal diseases, information pertaining to these key mechanisms of action is scarce, particularly for immune-mediated mechanisms of action. In the present work, we review the biochemical and molecular mechanisms of action of probiotics and synbiotics that affect the immune system.

## 1. Introduction

Pediatric intestinal diseases comprise a variety of clinically important conditions, such as infectious diseases (acute diarrhea, antibiotic-associated diarrhea (AAD), *Clostridium difficile*-associated diarrhea, and *Helicobacter pylori* infection), and necrotizing enterocolitis (NEC), as well as some non-communicable chronic diseases, including inflammatory bowel diseases (IBD) (ulcerative colitis and Crohn disease) and cystic fibrosis.

Pediatric infectious diseases are the most important illnesses in children, especially in preschool centers [1]. Children who go to daycare centers are at a 2.2–3.5-fold greater risk of developing gastrointestinal infections than children who stay at home [2,3]. In addition, studies have suggested that poor hygiene is related to the development of such infections [4,5].

Acute infectious diarrhea and AAD are the two primary manifestations of gastrointestinal pediatric infections. Acute diarrhea is frequently originated through viral infection, with rotavirus and Norwalk virus infections being common causes of gastroenteritis in children. In addition, important infectious bacteria are implicated in day care-associated gastrointestinal disorders, such as *Escherichia coli*, *Salmonella* sp., *Campylobacter jejuni*, *Clostridium difficile* and *Helicobacter pylori* [6,7,8,9].

In developing countries, more than 500,000 people die annually due to diarrhea associated with rotavirus gastroenteritis. In Europe, almost every child will have experienced an episode of rotavirus gastroenteritis, and one in 54 will need hospitalization [10].

AAD is known to disrupt the gastrointestinal microbiota that marks in a variety of medical symptoms. The AAD incidence in children in primary health services is approximately 10% [11,12]. *C. difficile* infections primarily occur in immunocompromised hosts and represent serious infection for which the primary treatment is antibiotic therapy. In fact, *C. difficile* infection is the leading cause related to antimicrobial therapy, accounting for nearly 15–25% of all AAD episodes [13]. 

Additional pediatric intestinal diseases that are frequently associated with intestinal dysbiosis include: NEC, a health condition that is principally appreciated in premature infants with bowel undergo necrosis [14]; ulcerative colitis, a chronic IBD of unknown etiology that is characterized by acute exacerbations of intestinal complications, followed by remissions; Crohn’s disease, a systemic disorder in which the development of host genetic susceptibility represents an important etiological factor [15]; and cystic fibrosis, a fatal genetic disease without cure, affecting the digestive system and lungs with some typical complications, such as difficulty digesting fats and proteins, malnutrition and vitamin deficiencies resulting from an inability to absorb nutrients, chronic infections and aberrant inflammation [16].

The current treatment for pediatric infectious diseases focuses on oral or intravenous rehydration, nutritional support, and in some cases, antibiotics. The new therapeutic alternatives, for example antiemetics, antidiarrheal agents, and probiotics are often proposed. Oral rehydration therapy prevents only related dehydration [17], but does not affect the frequency of bowel movements, diarrheal duration, or intestinal barrier function [18,19]. Indeed, to better limit and heal intestinal damage, new treatment alternatives are required.

Alteration to the gut microbiome through the administration of beneficial microbes, typically referred to as probiotics, is an active area of investigation [15,20,21,22]. Probiotics are defined as “live microorganisms that confer a health benefit to the host when administered in adequate amounts, although dead bacteria and bacterial molecular components may also exhibit probiotic properties” [22]. Recent reviews and meta-analyses have suggested an effect of probiotics in the treatment and prevention of gastrointestinal and upper respiratory infections in children [23,24,25,26,27,28,29,30]. Probiotics appear to have a beneficial impact in treating acute infectious diarrhea and reducing AAD. However, the potential advantage of probiotics in the prevention of traveler’s diarrhea and *C. difficile*-associated diarrhea, as well as the adverse effects in *H. pylori* eradication, NEC, IBD, and cystic fibrosis remain unclear, the principal reason being that the effects tend to be strain-specific [3,31,32,33].

A prebiotic is a non-viable food component that confers a health benefit to the host and is associated with the modulation of the intestinal microbiota. Using prebiotics and probiotics in combination is often described as synbiotics [34,35].

The administration of probiotics for the prevention and treatment of a variety of pediatric infectious diseases has received increasing attention worldwide. Many scientific reports from different societies, such as the European Society of Pediatric Gastroenterology, Hepatology and Nutrition (ESPGHAN) [24], the American Academy of Pediatrics [36], the World Gastroenterology Organization [37], and the Canadian Pediatric Society [38] have indicated the benefits of probiotics, supporting recommendations for the use of probiotics to treat acute gastroenteritis and for the reduction of AAD. Several important mechanisms that underlie the observed beneficial effects of probiotics include secretion of antimicrobial substances, competitive adherence to the mucosa and epithelium, strengthening of the gut epithelial barrier, and modulation of the immune system [22,23,32]. Probiotic effects that are mediated through these mechanisms are an important issue that needs to be addressed. In addition, pro-inflammatory transcription factors, cytokines, and apoptosis-related enzymes can also be affected by probiotic strains. However, information on the mechanism of action of these effects, which are mediated by probiotics, is scarce, especially details related to the modulation of the immune system.

Therefore, the present review was conducted to investigate what is known from published research on the immune-mediated effects of probiotics and synbiotics in the prevention and treatment of pediatric intestinal diseases, with a special focus being placed on the mechanisms of action related to immune system modulation.

## 2. Materials and Methods

A comprehensive search of the relevant literature was performed using electronic databases, including MEDLINE (PubMed), EMBASE, and the Cochrane Library. We searched for scientific articles published between 2009 and 2017 in English in MEDLINE through PubMed. We used the MeSH terms “probiotics” and “synbiotics” combined with “infection” “pediatrics” and “gastrointestinal diseases”. We evaluated the results that were obtained using the following equation search: ((“infection”[MeSH Terms] OR “infection”[All Fields]) AND (“pediatrics”[MeSH Terms] OR “pediatrics”[All Fields] OR “pediatric”[All Fields])) AND ((“probiotics”[MeSH Terms] OR “probiotics”[All Fields] OR “probiotic”[All Fields]) OR (“synbiotics”[MeSH Terms] OR “synbiotics”[All Fields] OR “synbiotic”[All Fields])). Our search yielded 211 articles, 31 of which were selected that specifically inform on probiotic mechanisms of action. Additionally, we searched the reference lists of the included articles for potential relevant literature.

## 3. Results and Discussion

### 3.1. Major Clinical Effects and Related Mechanisms of Action of Probiotics in Pediatric Intestinal Diseases

#### 3.1.1. Gastrointestinal Infections

One thousand and sixty-two preschool children were enrolled in a double-blind, randomized, controlled study to test the effects of *L. casei rhamnosus*, *L. rhamnosus* T cell-1, multiple probiotics, and a placebo over 3 and 7 month periods [39]. Single strain probiotic supplementation significantly decreased the incidence of bacterial infections. Nevertheless, the multiple probiotic supplements did not show any effect. The only strain that decreased infectious disease at 3 months was *L. casei rhamnosus*. The authors hypothesized that the results were mediated through the action of lactobacilli species, as they can affect antigen-specific IgG1/IgG2 antibodies and cytokine responses and can also stimulate dendritic cells (DC) and produce a Th1 response [39].

*L. rhamnosus* GG strain is the most evaluated probiotic on pediatric population, alone or in combination with prebiotics or vitamins. A prospective, randomized, double-blind, placebo-controlled trial was performed to test the *L. rhamnosus* GG administration during 3 months in 281 children. The *L. rhamnosus* GG treatment was only successful only in the prevention of upper respiratory tract infections in children who attended primary health services [40]. This study showed no significant effect of the *L. rhamnosus* GG treatment with respect to the observed number of gastrointestinal infections and the number of diarrhea and vomiting episodes. In contrast, using a similar methodology in hospitalized children, Hojsak et al., 2010 observed that a *L. rhamnosus* GG treatment significantly reduced the gastrointestinal infections, vomiting, and diarrhea episodes [41]. In both studies, the authors only mentioned a few documented effects of probiotics related to antimicrobial properties and enhancement in mucosal barrier and their immunomodulatory action. 

An immunological approach was performed in 124 children (82 infected with rotavirus and 42 with cryptosporidial diarrhea) that evaluated the *L. rhamnosus* GG effects on immune response and intestinal permeability. In the *L. rhamnosus* GG group less children had repeated diarrheal events and impaired intestinal function. Moreover, children that received *L. rhamnosus* GG have an increase in IgG levels post-intervention. *L. rhamnosus* GG administration exhibited a significant enhancement in intestinal permeability in children with cryptosporidial diarrhea [42]. The authors stated that the mechanism by which probiotics produce an immunomodulatory action is not completely understood and might be related to the modulation of immune responses (innate and adaptive), increasing serum IgG and secretory IgA to enteric pathogens, including *Salmonella typhi* and rotavirus [42].

Another study with *L. rhamnosus* GG was performed in 90 hospitalized children, and they received *L. rhamnosus* GG plus vitamin B, vitamin C, and zinc or placebo. The consumption of a combination of *L. rhamnosus* GG and micronutrients was effective in reducing the incidence of gastrointestinal infections and the length of hospitalization compared to placebo [43]. Furthermore, the duration and severity of symptoms were reduced [43]. No mechanism of action was reported to be associated with the observed effects.

The last *L. rhamnosus* GG study was a follow-up study that was conducted for 3 and 5 years in 109 and 96 children, respectively. Children received hydrolyzed protein formulas with *L. rhamnosus* GG, and the primary expected result (the decrease of the incidence of acute gastroenteritis mediated through the action of *L. rhamnosus* GG) was not observed at the analyzed time-points [44].

Studies on the administration of *L. rhamnosus* GG have shown contradictory results. In some studies, probiotic treatment decreased the frequency and gravity of gastrointestinal diseases, and in others, an effect was not observed. It is important to mention that the administration doses and time interventions were different in these studies, and future studies should be standardized to assess a potential successful result. Regarding the mechanism of action, the primary mechanism proposed for *L. rhamnosus* GG was the modulation of the innate and adaptive immune system, but this was mostly based on speculation.

The gastrointestinal effects and antibiotic sensitivity of *L. salivarius* CECT5713 were analyzed in 80 6-month-old children during 3 and 6 months of intervention. A probiotic treatment decreased the frequency of diarrhea and respiratory infections compared with placebo group [45]. Fecal concentration of butyric acid has augmented in *L. salivarius* CECT5713 group, this acid reduce the colonic pH and increase the peristaltic movements, promoting an advantageous environment for commensal bacteria. The authors observed a lower frequency of diarrhea in *L. salivarius* CECT5713 group, and they related this finding to in vitro assays, reporting on the production of antimicrobial compounds through *L. salivarius* CECT5713 action [45].

In 2012, 215 infants were enrolled to test the effects of *L. fermentum* CECT5716 plus galactooligosaccharides (GOS) during 6 months. *L. fermentum* administration was useful for the prevention of gastrointestinal infections in infants [46], increasing bifidobacteria and lactobacilli. The production of short chain fatty acids and IgA concentrations did not change during the study. The authors linked those changes in bifidobacteria and lactobacilli with the reductions in the number of gastrointestinal episodes observed in *L. fermentum* CECT5716 plus GOS group [46].

A follow-up study that lasted 3 years observed similar values of growth, frequency of infectious and non-infectious diseases in children that received the *L. fermentum* CECT5716 formula compared with placebo [47]. The proposed mechanism of action was the innate response activation through the *L. fermentum* CECT5716 plus GOS, although no changes in fecal IgA were detected [47]. The effects of *L. salivarius* CECT5713 were mediated through butyric acid production, and a *L. fermentum* CECT5716 plus GOS treatment appeared to produce changes in the intestinal microbiota.

Regular calcium content plus *L. casei* CRL431 or *L. reuteri* DSM17938 treatments were tested in 494 children. The frequency of diarrhea episodes was significantly lower in the *L. reuteri* group compared with the placebo group [48]. Similar results were shown in children supplemented with fermented milk with killed-*L. paracasei* CBA L74 or placebo for 3 months on a daily basis. The probiotic treatment decreased the number of episodes of acute gastroenteritis compared to placebo [49]. These effects were mediated by an augment of immunity peptides. Moreover, to their antimicrobial character these peptides regulate the T cells activity, DC, macrophages, monocytes, and neutrophils, as well as the production of secretory IgA, in the killed-*L. paracasei* CBA L74 group [49].

The administration of *L. casei* DN-114 001 was effective in decreasing gastrointestinal infections in 638 3–6-year-old children in daycare centers/schools [50]. The authors recognized that this trial studied a precise probiotic strain, dose, and age group, and their findings cannot be generalized for other species or consequences without explaining a mechanism of action.

Conversely, several studies did not show clinical effects of probiotic strains over gastrointestinal infections. *L. reuteri* DSM 17938 was administered in children, no differences were found between the probiotic and placebo groups [51]. In addition, healthy children over 4 months old were investigated in a study consisting of a 3-month product consumption period and a 1-month follow-up period with a fermented milk containing *L. casei* CNCM I-1518 [52]. These negative results were related to the administered doses (low doses), the strain specificity, and the intervention time of each study. Unfortunately, none of these studies evaluated the mechanism of action of the probiotics.

Members of the genus *Bifidobacterium* are another important group of probiotic species. *B. lactis* B94 plus inulin were tested in 79 children with diarrhea. Compared with 77 children in the placebo group, the synbiotic treatment reduced the length of diarrhea, and this reduction was most pronounced in the *Rotavirus* diarrhea cases [53]. The number of diarrheal stools on the third day was significantly smaller in the synbiotic group. Although the reduction in stool frequency on the third day was more pronounced, the number of diarrheal stools on the second day was also significantly smaller in the synbiotic group [53]. The authors stated that the presented effects were related to inulin administration. Although prebiotics are generally well tolerated, they can cause bloating, abdominal pain, and diarrhea when taken in excessive amounts. In this study, the patients were given 900 mg of inulin, and no symptoms of discomfort were observed [53]. Further studies in this field are still required to reveal the actual mechanism of action in a synbiotic treatment.

In contrast, with the previous study, the results of *B. animalis* subsp. *lactis* administration was tested in 727 children. The ingestion of probiotic strain failed to prevent gastrointestinal infections in children [54]. In another double-blind, placebo-controlled study with 109 children who were randomly divided into the receiving group being administered the same probiotic strain and the placebo group, the probiotic administration failed in decreasing the reported gastrointestinal symptoms or fever [55]. The authors hypothesized that xylitol, present in the administered tablets, might perform as a prebiotic ingredient to influence the gut colonization of probiotic strain. 

Finally, a recent study was conducted in 290 infants that received a mixture of *B. animalis* subsp. *lactis* and *L. rhamnosus* GG for a 6-month intervention period. The outcomes revealed that the mixture administration did not decrease the absence of children from primary health services [3]. It is important to mention that the use of products containing other probiotics and prebiotics were not prohibited during the study [3]. A potential immunoprotective effect of breastfeeding, might thus have reduced the study power. Molecular mechanisms of action were not listed in the study.

Several probiotics strains were administered in the aforementioned studies, but it appears that the intervention time and method used to evaluate the primary outcome are the most important variables to obtain promising results. *L. rhamnosus* GG was the most assessed strain, showing contradictory results in the incidence and severity of gastrointestinal diseases. The proposed mechanism of action of this bacterium was the modulation of the innate and adaptive immune system. Other strains, such as, *L. salivarius* CECT5713, act through butyric acid production, and an *L. fermentum* CECT5716 plus GOS treatment generated a number of changes in the intestinal microbiota. In addition, some studies did not show clinical effects of the probiotic strains over gastrointestinal infections, even the strains belong to the *Lactobacillus* or *Bifidobacterium* genera. Table 1 shows the most relevant information regarding selected studies of children with probiotic approaches. 

#### 3.1.2. Antibiotic-Associated Diarrhea (AAD)

The AAD happens when antibiotics disrupt the natural ecology in the mucosal tract, causing the increase of pathogens bacteria. The symptoms of AAD include abdominal pain and extensive bowel movements [56]. 

A recent study was performed in 97 children to test the effectiveness of *L. reuteri* DSM 17938 in the prevention of AAD. The probiotic administration did not change the frequency or rigorousness of AAD [57]. A weakness of this study was the lack of fecal analysis to confirm compliance and survival of the probiotic administration.

Twenty-three studies with 3938 participants were included in a systematic review of the treatment of AAD with probiotics. Analyzed trials included treatment with either *Bacillus* spp., *Bifidobacterium* spp., *Clostridium butyricum*, lactobacilli, *Lactococcus* spp., *L. cremoris*, *Saccharomyces* spp., or *Streptococcu*s spp., alone or in combination. Two strains (*L. rhamnosus* or *S. boulardii*) may be the most recommended. In the case of immunocompromised or debilitated children, the use of probiotics needs a rigorous evaluation to assess patient safety [56].

Finally, the authors gave some evidence that proposes a defensive effect of probiotics in AAD. For that reason, further well-designed studies are desired to adjust the safety of probiotics as a treatment option for AAD. Currently, probiotic administration in the pediatric population with AAD is avoided. No specific mechanisms of action have been addressed in most of studies related to treatment of ADD with probiotics. 

#### 3.1.3. *Clostridium difficile*-Associated Diarrhea

*Clostridium difficile*-related diseases include severe diarrhea, colitis, and pseudomembranous colitis. The treatment is costly, and probiotics have been suggested as a cheap strategy to both prevent and treat *C. difficile*-associated diarrhea. In the last systematic review, a total of 31 studies with 4492 participants were discussed regarding this issue. The administration of probiotics was safe and effective for preventing *C. difficile*-associated diarrhea [58]. In contrast to AAD, probiotic administration seems to be safe, well tolerated, and indicated as coadjuvant therapy in *C. difficile*-associated diarrhea. In addition, immunocompromised or severely debilitated patients as a risk group always require a risk-benefit evaluation. Further clinical trials are needed to elucidate the mechanisms by which probiotics prevent *C. difficile*-associated diarrhea [58].

#### 3.1.4. *Helicobacter pylori* Gastritis and Peptic Ulcer

*Helicobacter pylori* is accepted as a main etiological issue in the pathogenesis of gastritis and peptic ulcer disease. In pediatric population, the *H. pylori* eradication has a failure percentage of more than 30%, due to reduced compliance, antibiotic resistance, and the incidence of adverse events. A study was performed in sixty children with *H. pylori* who treated with *H. pylori* eradication treatment protocol (omeprazole + amoxycillin + furazolidon or other antibiotic), and randomly divided to receive either probiotic mixture or placebo. The probiotic treatment with *L. acidophilus*, *L. rhamnosus*, *L. bulgaricus*, *L. casei*, *S. thermophilus*, *B. infantis*, and *B. breve* increased the *H. pylori* eradication ratio. Moreover, it was the most effective treatment in lowering the frequency of nausea, vomiting and diarrhea [59]. The authors argued that the observed probiotic effects might have been mediated by *Lactobacillus* strains that interfered with the activity of *H. pylori* through preventing its adherence to epithelium and incapacitating its primary virulence factor, urease enzyme. 

Developing countries have a higher prevalence of *H. pylori* infections. Therefore, new non-invasive therapies are preferred, among which the *H. pylori* stool antigen testing is included. Twenty-eight children with a positive stool test for *H. pylori* were randomized in a clinical trial, with individuals receiving *S. boulardii* or placebo for one month. The probiotic administration reduced the mean amount of antigen present during the study, but was not competent of causing the *H. pylori* abolition when used as a mono-therapy [60].

These results contrast with another study in which children who had biopsy-proven *H. pylori* infections were randomly divided to receive the *H. pylori* eradication treatment protocol plus *B. lactis* B94 and inulin for 14 days, and the standard therapy alone. The abolition ratio were similar in both groups. In addition, the synbiotic do not show advantage compared with standard therapy conducted alone [61]. The limitations of this study were that *H. pylori* culture and antibiotic susceptibility tests were not performed, *B. lactis* B94 colonization in the feces was not evaluated, and the authors stated that the sample size was relatively small. In addition, the authors only mention and speculate that the probiotic effects might be achieved by the production of short-chain fatty acids, autolysins, mucin, and bacteriocins and/or by the binding of some specific strains to the same glycolipid receptors as *H. pylori* [61].

Recently, Feng et al., 2017 described some important results from 29 trials involving 17 different probiotic treatments. When the standard therapy was accompanied with a probiotic strain, the eradication of *H. pylori* was successful. The most identified strain in the aforementioned effect was *L. casei* as mono-therapy, as a multi-strain therapy *B. infantis*, *B. longum*, *L. acidophilus*, *L. casei*, *L. plantarum*, *L. reuteri*, *L. rhamnosus*, *L. salivarius*, *L. sporogenes*, and *S. thermophilus* was the best in reducing the incidence of diarrhea. Indeed, probiotic ingestion is suggested to supplement *H. pylori* eradication treatment protocol in pediatric population, and the effectiveness of this therapy is related with the particular probiotic administration. Finally, the proposed mechanism of actions for *H. pylori* eradication include the following: increased competition with *H. pylori* to bind surface receptors of intestinal epithelial cells; inhibiting adhesion of *H. pylori* to mucosa; altering the inflammatory factors expression; strengthening of the intestinal mucosa barrier; and secretion of antimicrobial substances [62].

Taking into consideration the current literature, AAD is the only pathology for which probiotics should be avoided. Likewise, the mechanism of action and their clinical effects requires further investigation. Moreover, the administration of probiotics was a safe coadjuvant therapy in the case of *C. difficile*-associated diarrhea.

#### 3.1.5. Necrotizing Enterocolitis

Necrotizing enterocolitis (NEC) is a disturbing inflammatory disorder that primarily occurs in preterm neonates and causes high mortality rates (20–30%) [63]. In fact, NEC is the leading cause of death from gastrointestinal disease in premature newborns with low birth weight (LBW). Probiotics were recently reported to be beneficial to infants with NEC. Indeed, several meta-analyses indicated that probiotics reduce the risk of NEC and all causes of mortality, but not of sepsis, in preterm infants [14]. Such a treatment has also been shown to decrease mortality and days of hospitalization and to increase the effectiveness of exclusive enteral nutrition in the days following treatment [64,65]. 

The pathogenesis of NEC is still discussed, although besides prematurity and LBW, known risk factors include early formula feeding and an altered intestinal microbiota [66]. In 2010, Alfaleh et al., 2010 established that enteral supplementation with probiotics reduces the risk of severe NEC and mortality in preterm newborns [67]. Twenty-four trials showed that enteral probiotic supplementation significantly decreased the incidence of severe NEC and mortality. Probiotic preparations containing either *Lactobacillus* alone or in combination with *Bifidobacterium* were found to be positive. 

*Lactobacillus rhamnosus* HN001 is a Gram-positive bacterium which is beneficial for inflammatory diseases treatment due to its probiotic actions [66]. Though the specific mechanisms of action of that probiotic are still unknown related to NEC, different studies, with strains such as *L. rhamnosus* HN001, have reported that the microbial DNA receptor Toll-like receptor-9 (TLR9) can be activated, which has been described as a potential therapeutic target. Thus, *L. rhamnosus* HN001 is capable of attenuating NEC in in vitro studies through via microbial DNA (Lr-DNA). Such protection requires activation of TLR9, which has no evidence of toxicity [68]. 

*Lactobacillus reuteri* is a probiotic bacterium which has also been studied with the aim to prevent NEC. This probiotic inhibits enteric infections and controls the immune system. *L. reuteri* produces a potent antibacterial compound that inhibits the growth of microorganisms and modulates tumor factor necrosis alpha (TNF-α) synthesis from bacterial lipopolysaccharide (LPS)-activated monocytoid cells [69,70,71]. The human-derived *L. reuteri* strains DSM17938, ATCC PTA4659, ATCC PTA 5289, and ATCC PTA 6475 decreased the LPS induced-inflammation in small intestinal epithelial cells and in the ileum of neonatal rats [71]. Afterwards, Liu et al. (2012) reported that those *L. reuteri* strains reduced bowel inflammation by downregulating interleukin (IL)-6, TNF-α, TLR4, and nuclear factor κ-B (NF-κB) and upregulating IL-10 in newborn rats with NEC. In addition, *L. reuteri* led to a decrease in intestinal TLR4, TNF-α and IL1β in the experimental model, demonstrating a potential therapeutic profile of this probiotic in the prevention of NEC [72].

With respect to NEC, it has been described that a live probiotic from diet might remain effective at reducing the translocation of pathogens in a short-term animal model [73]. Copeland et al. (2009) studied whether a live probiotic diet, such as *L. lactis*, could have the same effects in a long-term neonatal rabbit model. The fortified probiotic diet produced a significant reduction in translocation of *Enterobacter* to the liver and the colonization in the stomach and lungs was also lower in rabbit pups. Moreover, colonization or translocation of the probiotic outside of the gastrointestinal tract was prevented by the diet and rabbits tested positive for *L. lactis* in the cecum, exhibiting the ability of this probiotic to survive the transit to the colon [66].

Immunity and subsequent resistance to enteric pathogens of gut predominate during initial infancy and do not appear during any other stage of life [74]. Thus, infections caused by enteric bacterial pathogens are one of the most important causes of severe infantile diarrhea. Likewise, it has been reported that probiotic treatments, such as *Lactobacillus acidophilus*, attenuate bacterial-mediated intestinal injury and inflammation, which enhanced the host defense against enteric bacterial infection [75]. Foye et al. (2012) showed that early inoculation of the probiotic *L. acidophilus* might improve host-protective immunity to enteric bacterial pathogens by means of the TGF-β (transforming growth factor b) response. Thus, the anti-inflammatory effects were triggered through decreasing Smad 7 expression, allowing TGF-β to activate IκB-α and lower NF-κB accumulation [76]. An in vivo study was carried out to assess the probiotic properties of *L. acidophilus*, a prebiotic, inulin or both (synbiotic) on pathogen-induced inflammatory reactions in neonatal mice. Mice were inoculated twice per week for 4 weeks with *L. acidophilus*, inulin, or the synbiotic and were challenged with the pathogenic bacterium *C. rodentium* at 5 weeks. They observed that *L. acidophilus* and/or prebiotic inulin consumption reduced *C. rodentium*-induced early morbidity and inflammation in mice. In addition, an in vitro study was carried out in mice. The intestinal epithelial cell line CMT-93 was treated with *C. rodentium* to determine changes in NF-κB and Smad (similarity to the *Drosophila* gene Mothers Against Decapentaplegic (Mad)) 7 levels. Thus, NF-κB was activated at 60 min post-*C. rodentium* infection, as indicated by IκB-α degradation in CMT93 cell line. These findings indicated that TNF-α production reveals that *C. rodentium* bacteria-induced NF-κB activation and Smad 7 response was associated with the pro-inflammatory cytokine production in intestinal epithelial cells. This study supports the fact that probiotics are capable of promoting host-protective immunity and of attenuating *C. rodentium*-induced bowel inflammation through mechanisms that affect NF-κB and Smad 7 expression [76]. 

Probiotics reduce the pro-inflammatory status by immunomodulation and by protecting tissues against microbial infection [77], and their mechanism of action consists of modifying the production of cytokines in diverse cell populations. *Lactobacillus rhamnosus* GG is a probiotic strain that is commonly integrated into fermented products. In rats, it has been demonstrated to decrease LPS-induced systemic inflammation [78]. Accordingly, it has been described that *L. rhamnosus* GG provokes IL-4, IL-10, and urocortin expression and inhibits LPS-induced TNF-α in trophoblast cells from human term placenta. Thus, these findings support the immunomodulatory effect of probiotics in human placenta [79,80]. Another study reported that the probiotic *L. rhamnosus* GG diminishes *Campylobacter jejuni* infection and butyrate transporter and receptor are expressed in differentiated Caco-2 cell monolayers. The butyrate protection against *C. jejuni* adhesion are correlated to the existence of HCAR2 and SLC5A8, which are a receptor and transporter of butyrate, respectively. Moreover, the *L. rhamnosus* GG exerts the same effects [81]. Concerning inflammatory cytokine production, other described mechanisms include TGF-β/SMAD and NF-κB signaling pathway. The probiotic *L. acidophilus* was reported to decrease *Salmonella*-induced NF-κB activation in human intestinal Caco-2 cells. Moreover, TNF-α and IL-8 expression was significantly lowered and TGF-β1 and MIR21 levels were higher in *L. acidophilus*-treated cells compared with cells infected with only *Salmonella* [82]. In contrast, the levels of SMAD7, which it is a target of MIR21, were lower in cells treated with *L. acidophilus* or synbioticaly with inulin. Indeed, consistent with TGF-β1/MIR21 and SMAD7 expression, transcriptional activity of SMAD3/4 was significantly increased in cells treated with *L. acidophilus* or synbiotics. This suggests that TGF-β1/MIR21 expression might be useful as a marker to assess the anti-inflammatory effects of different *Lactobacillus* strains and that probiotics may be a new treatment approach for inflammation due to *Salmonella* infection [82].

Finally, Rojas et al., 2012 evaluated the use of prophylactic probiotics to prevent death and nosocomial infections in preterm newborns. Although they observed a 40% decrease in the incidence of NEC in the group treated with probiotics, they did not observe a primary outcome of the study. However, it may be clinically relevant and it is consistent with others studies that assessed NEC [83].

In summary, animal studies appear to be more widely used in the evaluation of potential mechanisms of actions for probiotics. The results from these studies show that probiotic treatment might improve inflammatory status by immunomodulation, treating and reducing NEC. Further analyses are required in human trials to ensure that no adverse effects occur from the treatment of probiotics. Table 2 shows the primary information relating to the mechanism of action in NEC. 

#### 3.1.6. Inflammatory Bowel Diseases

Ulcerative colitis and Crohn’s disease are the primary manifestations of IBD. Recently, we published a review that focuses on the treatment of chronic diseases in in vitro, animal, and human studies after the probiotics treatment. The use of probiotic strains seems to be potentially well tolerated, effective, and safe in patients with IBD. Indeed, probiotics improved clinical symptoms in patients with mild to moderate active ulcerative colitis; the results in Crohn’s disease are unclear. Some probiotics and their supernatants act by decreasing the pro- and inflammatory cytokines gene expression by the modulation of TLR, NF-κB, and mitogen-activated protein kinase (MAPK) pathways. Importantly, there is no recommendations of any probiotics strain for treatment of Crohn’s disease in children. Additionally, probiotics definitely seem more favorable for ulcerative colitis, where some strains have previously confirmed to be effective [15].

#### 3.1.7. Cystic Fibrosis

Intestinal inflammation is a common symptom in patients with cystic fibrosis, in whom bacterial overgrowth may also be present. Younger patients with cystic fibrosis might be good candidates for supplementation with probiotics, because their intestinal microbiota is often abnormal due to immense exposure to antibiotics, suggesting the disturbance of intestinal barrier function and the dysregulation of innate immune mediators [16]. A prospective randomized, double-blind, placebo-controlled trial was carried out in 61 children with cystic fibrosis to evaluate *Lactobacillus reuteri* ATCC55730 in altering the degree of respiratory exacerbations and of infections of upper respiratory and gastrointestinal tracts. Pulmonary exacerbations were significantly decreased in the probiotic group. Probiotic and control groups did not significantly change in the mean number and duration of hospitalizations as a consequence of pulmonary exacerbations, gastrointestinal infections, fecal calprotectin concentration, and tested cytokines (TNF-α and IL-8). The authors concluded that probiotic administration attenuates pulmonary exacerbations in cystic fibrosis patients with mild-to-moderate lung disease, and the mechanistic speculation of those results was that *Lactobacillus* strains effect immune responses beyond the intestinal tract [16].

Based on the complexity of cystic fibrosis, the probiotic treatment requires further and detailed investigation to ensure a safe therapy.

#### 3.1.8. Other Studies

Recently, our research group has reported different in vitro and in vivo studies related to probiotics. Thus, we examined the anti-inflammatory properties of probiotics in human DC generated from CD34+ progenitor cells (hematopoietic stem cells) collected from umbilical cord blood that showed surface antigens of dendritic Langerhans cells, resembling to the lamina propria DCs in the intestine [84,85,86]. We incubated these intestinal-like human DCs with *Lactobacillus paracasei* CNCM I-4034, *Bifidobacterium breve* CNCM I-4035, *L. rhamnosus* CNCM I-4036 or its cell-free supernatants (CFS), *Salmonella typhi* CECT 725, *E. coli* CECT 742, CECT 515, and CECT 729 or a mixture of these treatments for 4 h. These probiotic treatments provoked an upregulation of TLR-9, toll-interacting protein, and CASP8 gene expression. Probiotic supernatants diminished pro-inflammatory cytokines and chemokines in DCs that were challenged with *S. typhi* and restored TGF-β levels in the existence of *S. typhi*. In addition, supernatants enhanced innate immunity due to the activation of TLR signaling, especially TLR-9, TLR-2, and TLR-4 gene expression [84,85,86].

Other results from our research group using an experimental model of obesity in Zucker rats and in human healthy volunteers who received selected probiotic strains have documented a number of immunomodulatory effects. Administration of *B. breve* CNCM I-4035 produced a significant increase in fecal secretory IgA content. IL-4 and IL-10 were up-regulated, whereas IL-12 was lower in the serum of subjects after the treatment with any of the three strains. Serum TNF-α levels diminished in Zucker-Lepr^fa/fa^ rats treated with *B. breve*, *L. rhamnosus*, or the mixture, whereas *L. paracasei* feeding showed a reduction of IL-6 levels in the serum of Zucker-Lepr^fa/fa^ rats. Moreover, probiotic administration downregulated the gene and protein expression of *Adamdec1* and *Ednrb*, and that of *Ptgs1/Cox1* at the gene expression level. This result was partially mediated by a reduction in both macrophage and dendritic cell populations [21,22,77,87,88]. Additionally, we have studied the early administration of *L. fermentum* CECT5716, which is a probiotic strain added in infant formula, in children. We reported that this probiotic preparation was safe and it did not produce quantifiable differences in children compared with the control group, but no specific mechanism of action was addressed [47,89]. 

In line with our findings, Tsilingiri et al. (2012) have developed an in vitro model system that offers several physiological characteristics that can be representative of a mucosal microenvironment, containing the existence of an organized mucus layer and an apical to basolateral polarity. The authors evaluated the effects of *L. paracasei* supernatant against *S. typhimurium* in healthy and IBD tissue [90]. They concluded that probiotics could be more appropriately used in patients in remission and not for the period of the acute phase of the disease. Additionally, the use of supernatant might be an effective and safe alternative for the treatment of acute IBD. This effect was observed in the co-incubation treatment, mediated through the abrogation of TNF-α release without affecting IL-10 secretion [90]. Moreover, in a well-conducted in vitro study, Buccigrossi et al. (2014) have showed the effects of *S. boulardii* supernatant in cells infected with rotavirus. The aforementioned supernatant prevents oxidative stress produced by rotavirus infection, and inhibits chloride secretion in Caco-2 cells [91]. These findings suggest that probiotics and their supernatants can exhibit important molecular effects.

**Figure 1:**
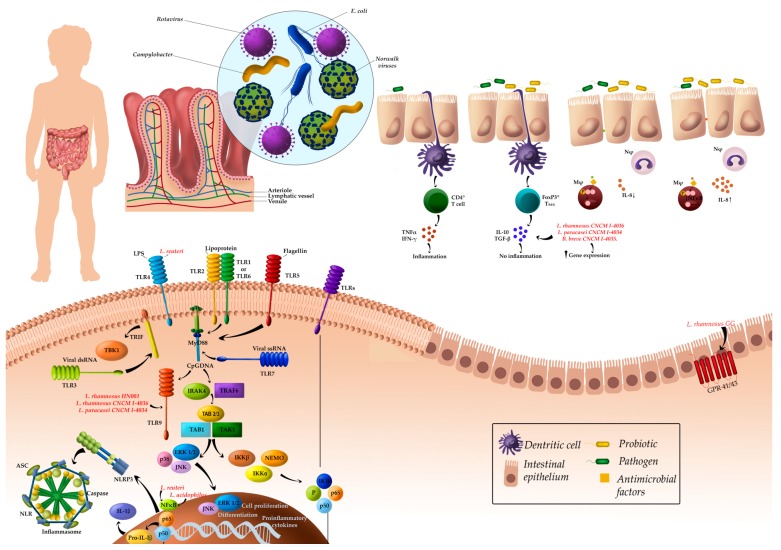
General probiotic mechanism of action in pediatric gastrointestinal infections. Abbreviations: ASC, apoptosis-associated speck-like protein containing a CARD; ERK, extracellular regulated kinase; IKK, IκB kinase; IL, interleukin; IFN, interferon; IRAK4, IL-1 receptor-associated kinase 4; JNK, Jun N-terminal kinase; NF-κB, nuclear factor κ-B; NEMO, NF-κB essential modulator; TNF-α, tumor factor necrosis alpha; TLR, toll-like receptor, TAB1/2/3, TAK binding proteins; TAK1, ubiquitin-dependent kinase of MKK and IKK; TBK1, serine/threonine-protein kinase 1; TGF, transforming growth factor; TRAF6, Tumor necrosis factor receptor-associated factor 6.

Finally, Figure 1 shows the general probiotic mechanism of action in pediatric intestinal diseases. Concerning gastrointestinal infections, the most important mechanism of action reported was the modulation of the immune system. With respect to NEC, the mechanism of actions are related to the TLR-signaling pathway and the butyric receptor.

## 4. Conclusions

Probiotics and synbiotics have been extensively studied in the evaluation of potential treatments for different gastrointestinal infections in children, such as acute gastroenteritis, AAD, *Clostridium difficile*-associated diarrhea, *Helicobacter pylori* gastritis, and peptic ulcer, as well as in other intestinal pathologies associated with gut dysbiosis. It appears that probiotics and synbiotics may be useful in improving such pathologies, except for AAD and Crohn’s disease. In any case, major adverse effects of probiotics have since been reported. Although such effects are supported by numerous clinical studies, further research is required to corroborate the adequate doses and time of treatment. Some strains, such as *L. rhamnosus*, have been reported for the treatment of pediatric infections, despite the existence of other potential probiotics that should be studied. Immune-mediated mechanisms of action of probiotics include the modulation of both innate and adaptive immunity. However, most of the reported studies only made speculations and did not attempt to evaluate specific biomarkers of systemic or intestinal immunity. Only a few in vitro and animal studies have shown that modulation of the immune system can be mediated through the interaction of probiotics with intestinal TLR, which in turn affects inflammatory cascade signaling, expression of cytokines and some intestinal host genes involved in inflammation. Thus, there is a need for designing further studies using probiotics and synbiotics in pediatric intestinal diseases and addressing their potential mechanisms of action though appropriate biomarkers of immunity and inflammation to support and provide scientific reasons that are able to explain the clinical benefits of specific probiotic strains.

## Figures and Tables

**Table 1:** Prevention and treatment of pediatric gastrointestinal infections with probiotics.

Reference	Participants	Probiotic Strain/Treatment	Time	Primary Outcome
Song-Lin et al., 2009 [39]	986 children	*L. casei rhamnosus*, *L. rhamnosus* T cell-1, and a mixture of strains	7 months	*L. casei rhamnosus* reduced respiratory infections, whereas multiple probiotic supplementation reduced the gastrointestinal disease. *L. rhamnosus* T cell-1 decreased the incidence of bacterial infection at 7 months
Hojsak et al., 2010 [40]	281 children	*L. rhamnosus* GG	3 months	Only prevention of upper respiratory tract infections
Hojsak et al., 2010 [41]	742 children	*L. rhamnosus* GG	1 week	*L. rhamnosus* GG treatment significantly reduced the risk for gastrointestinal infections, vomiting, and episodes of gastrointestinal infections
Kulandaipalayam et al., 2014 [42]	124 children	*L. rhamnosus* GG	1 month	*L. rhamnosus* GG decreased diarrheal episodes and restored normal intestinal permeability
Bruzzese et al., 2016 [43]	90 children	*L. rhamnosus* GG plus vitamins B, C and zinc	2 weeks	Treatment reduced incidence of gastrointestinal infections and length of hospitalization
Maldonado et al., 2010 [45]	80 children	*L. salivarius* CECT5713	6 months	*L. salivarius* CECT5713 decreased incidence of diarrhea and respiratory infections
Maldonado et al., 2012 [46]	215 children	*L. fermentum* CECT5716 plus GOS	6 months	Synbiotic administration prevented community-acquired gastrointestinal infections in infants
Maldonado et al., 2015 [47]	91 children	*L. fermentum* CECT5716 plus GOS	3 years follow-up	All variables measured were similar compared with placebo
Scalabrin et al., 2017 [44]	109 children	*L. rhamnosus* GG	5 years follow-up	A decrease in the incidence of acute gastroenteritis was not detected
Agustina et al., 2012 [48]	494 children	RCC plus *L. casei* CRL431, or RCC plus *L. reuteri* DSM17938	6 months	Incidence of all reported diarrhea and diarrhea incidence in children with a lower nutritional status were significantly lower in the *L. reuteri* group
Corsello et al., 2017 [49]	126 children	*L. paracasei* CBA L74	3 months	Probiotic treatment decreased the number of episodes of acute gastroenteritis
Merenstein et al., 2010 [50]	638 children	*L. casei* DN-114 001	3 months	*L. casei* DN-114 001 decreased gastrointestinal infections
Wanke et al., 2012 [51]	106 children	*L. reuteri* DSM 17938	1 week	No effects
Prodeus et al., 2016 [52]	599 children	*L. casei* CNCM I-1518	3 months	No effects
Islek et al., 2014 [53]	156 children	*B. lactis* B94 plus inulin	1 week	Synbiotic treatment decreased the duration of diarrhea
Hoksak et al., 2015 [54]	727 children	*B. animalis* subsp. lactis BB-12	1 week	No effects
Taipale et al., 2016 [55]	67 children	*B. animalis* subsp. lactis BB-12	2 years follow-up	No effects
Laursen et al., 2017 [3]	290 children	*B. animalis* subsp. lactis and *L. rhamnosus* GG	6 months	No effects

Abbreviations: GOS, galactooligassacharides; mixture of strains, three bifidobacteria, seven lactobacilli, *S. thermophilus*, and *E. faecium*; RCC, regular calcium content.

**Table 2:** General probiotic mechanisms of action in NEC.

Reference	Animal Species	Probiotic Strain/Treatment	Type of Study	Time	Primary Outcome
Good et al., 2014 [68]	Newborn mice/premature piglets	*L. rhamnosus* HN001	In vivo and ex vivo	5 days	*L. rhamnosus* HN001 or its DNA could protect against the development of NEC in animals. This seems to require DNA receptor TLR9 activation
Liu et al., 2012 [72]	Newborn rats	*L. reuteri*	In vivo and ex vivo	3 days	*L. reuteri* strains reduced intestinal inflammation by down-regulating the IL-6, TNF-α, TLR4, and NF-kB and up-regulating the IL-10 in rats with NEC
Liu et al., 2010 [71]	Newborn rats	*L. reuteri*	In vivo and in vitro (IPEC-J2 intestinal cell line)	3 days	*L. reuteri* reduced the inflammation caused by LPS in intestinal epithelial cells and in the ileum.
Copeland et al., 2009 [66]	Neonatal rabbit model	*L. lactis*, *E. cloacae*	In vivo	7 days	*E. cloacae* probiotic fortified diet was effective by reducing the colonization of pathogenic bacterium
Foye et al., 2012 [76]	Newborn mice	*L. acidophilus*	In vivo and in vitro (mouse intestinal epithelial cell line	7 weeks	*L. acidophilus*, inulin, or synbiotic attenuate *C. rodentium*-induced intestinal inflammation through NF-κB and Smad 7 expression
Bloise et al., 2010 [80]		*L. rhamnosus GG*	In vitro (primary trophoblast cells from human placenta)	3 h	*L. rhamnosus* GG provokes IL-4, IL-10 and urocortin expression and inhibits LPS-induced TNF-α in trophoblast cells from human term placenta

Abbreviations: NEC, necrotizing enterocolitis; IL, interleukin; LPS, lipopolysaccharide; NF-κB, nuclear factor κ-B; TNF-α, tumor factor necrosis alpha; TLR, toll-like receptor.
